# Supplementary material for: Analysis of the Physical Characteristics of an Anhydrous Vehicle for Compounded Pediatric Oral Liquids
Source: Pharmaceutics. 2023 Nov 20;15(11):2642. doi: 10.3390/pharmaceutics15112642 (PMC10674891; doi:10.3390/pharmaceutics15112642)
Supplement: Supplementary file 1 [file pharmaceutics-15-02642-s001.zip › pharmaceutics-2614755-supplementary.pdf]

Table S1: Summary of Experimental Methods and Their Purposes in Vehicle Development for Oral Suspensions

Experimental Method	Purpose and Importance
**Osmolarity Measurement**	Ensuring that the vehicle meets desired osmolarities. Oral solutions with high osmolarity can cause GI upset, diarrhea or even damage the GI tract (in newborns). Special attention to osmolality should be given in cases of administration via feeding tubes to the stomach or directly into the intestine. It can also affect the taste and texture of suspensions.
**Viscosity Analysis and Thixotropic Properties Analysis**	It has a direct effect on the ability of the suspension to maintain a uniform distribution of the active ingredient, thus affecting dose uniformity. High viscosity bases contribute to the sedimentation stability of the suspension. A base with the right viscosity can improve mouthfeel and palatability. Thixotropic bases, which are viscous at rest but flow more freely when agitated, offer the best - ease of preparation, sedimentation stability and ease of administration.
**pH Measurement**	The pH of a suspension can greatly affects the stability, solubility of the suspended particles and the interaction between different components in multi-component systems. The pH can also affect the taste of the suspension (not applicable for anhydrous suspensions).
**Sedimentation Analysis**	Sedimentation analysis provide information on the physical stability of an oral suspension, ensuring uniform dose delivery. Important is to prove redispersibility properties of suspension. Inconsistent doses due to sedimentation variability could compromise the efficacy and safety of the medication.
**Particle Size Evaluation**	To ensure even drug particle distribution and sedimentation stability, the desirable particle size in suspensions should range from 1 to 50 μm. An increase in particle size could lead to inaccurate dosing, deterioration of sedimentation stability, as well as affecting the texture and taste of the suspension.
**Droplet Size After Dispersion in Simulated Gastric Fluid**	This characteristic is particularly studied in relation to the emulsions and self-emulsifying drug delivery systems (SEDDS), which are capable of forming a spontaneous emulsion upon contact with aqueous solutions. The droplet size can influence the available surface area for absorption, the physical stability of the formulation, and the distribution of the API in the GI tract.
**Content Uniformity Test**	The content uniformity test is an essential quality control measure for oral suspensions and plays a critical role in ensuring the safety, efficacy, and reliability of the medication.
**Taste Masking Assessment**	To evaluate organoleptic properties and taste masking capabilities. This is particularly important for paediatric oral suspensions or suspensions for adults who have difficulty swallowing tablets or capsules.

Table S2: Chromatographic Conditions for Content Uniformity Analysis of Anhydrous Suspensions with Various APIs

API	Ketotifen	Metronidazole	Chloroquine Phosphate	Doxycycline	Enrofloxacin	Phenoxybenzamine Hydrochloride	Tretinoin	Nifedipine
**Column**	Acquity UPLC BEH C18 1.7 um 2.1 x 100 mm		Acquity UPLC BEH C18 1.7 um 2.1 x 50 mm					Acquity UPLC CSH Phenyl-Hexyl 1.7 um 2.1 x 100 mm
**Column Temp**	30°C	65°C	65°C	25°C	65°C	65°C	55°C	55°C
**Flow Rate**	0.4 mL/min	0.5 mL/min	0.8 mL/min	0.7 mL/min	0.8 mL/min	0.9 mL/min	0.7 mL/min	0.5 mL/min
**Injection Vol**	1 uL	1 uL	1 uL	1 uL	1 uL	1 uL	1 uL	1 uL
**Run Time**	6.0 min	3.0 min	3.0 min	4.0 min	3.0 min	4.0 min	4.0 min	9.0 min
**Delay Time**	0.0 min	0.0 min	0.0 min	0.0 min	0.0 min	0.0 min	1.0 min	0.0 min
**Mobile Phase A**	0.1% TFA in water							
**Mobile Phase B**	0.1% TFA in acetonitrile							
**Detection Wavelength**	300 nm	279 nm	343 nm	350 nm	317 nm	268 nm	355 nm	336 nm
**Target Concentration**	25 ug/mL	50 ug/mL	20 ug/mL	25 ug/mL	25 ug/mL	100 ug/mL	100 ug/mL	100 ug/mL
**Diluent**	Methanol	Methanol	Water	Methanol	Methanol	0.1% TFA in methanol	Methanol	Methanol

Table S3: Gradient Profiles of the Methods for Various APIs

API	Mobile Phase A	Mobile Phase B	Time, min - Gradient (Mobile Phases A:B, %)
**Ketotifen**	0.1% TFA in water	0.1% TFA in acetonitrile	0,00 min - 90%:10%;<br>0,50 min - 70%:30%;<br>3,50 min - 70%:30%;<br>5,00 min - 20%:80%;<br>5,10 min - 90%:10%;<br>6,00 min -90%:10%
**Metronidazole**	0.1% TFA in water	0.1% TFA in acetonitrile	0,00 min - 95%:5%;<br>1,00 min - 45%:55%;<br>2,00 min - 5%:95%;<br>3,00 min - 95%:5%
**Chloroquine Phosphate**	0.1% TFA in water	0.1% TFA in acetonitrile	0,00 min - 97%:3%;<br>2,00 min - 45%:55%;<br>3,00 min - 97%:3%
**Doxycycline**	0.1% TFA in water	0.1% TFA in acetonitrile	0,00 min - 90%:10%;<br>2,60 min - 70%:30%;<br>2,80 min - 10%:90%;<br>3,00 min - 90%:10%;<br>4,00 min - 90%:10%
**Enrofloxacin**	0.1% TFA in water	0.1% TFA in acetonitrile	0,00 min - 95%:5%;<br>1,00 min - 45%:55%;<br>1,10 min - 5%:95%;<br>2,00 min - 5%:95%;<br>2,10 min - 95%:5%;<br>3,00 min - 95%:5%
**Phenoxybenzamine Hydrochloride**	0.1% TFA in water	0.1% TFA in acetonitrile	0,00 min - 90%:10%;<br>3,00 min - 30%:70%;<br>4,00 min - 90%:10%
**Tretinoin**	0.1% TFA in water	0.1% TFA in acetonitrile	0,00 min - 80%:20%;<br>0,50 min - 65%:35%;<br>1,50 min - 65%:35%;<br>3,90 min - 0%:100%;<br>4,00 min - 80%:20%
**Nifedipine**	0.1% TFA in water	0.1% TFA in acetonitrile	0,00 min - 75%:25%;<br>6,00 min - 60%:40%;<br>6,01 min - 5%:95%;<br>6,60 min - 5%:95%;<br>6,61 min - 75%:25%;<br>9,00 min - 75%:25 %
